# Brain hemodynamic changes in amnestic mild cognitive impairment measured by pulsed arterial spin labeling

**DOI:** 10.18632/aging.102888

**Published:** 2020-03-12

**Authors:** Xiangbin Wang, Ding Ding, Qianhua Zhao, Xiaoniu Liang, Ling Peng, Xiaohu Zhao, Qian Xi, Zhang Min, Wei Wang, Xiaowen Xu, Qihao Guo, Pei-Jun Wang

**Affiliations:** 1 Department of Radiology, Tongji Hospital, Tongji University School of Medicine, Shanghai 200065, PR China; 2 Institute of Neurology, Huashan Hospital, Fudan University, Shanghai 200040, PR China; 3 Department of Radiology, Shanghai Liqun Hospital, Shanghai 200333, PR China; 4 Department of Radiology, The Fifth People's Hospital of Shanghai, Fudan University, Shanghai 200240, PR China; 5 Department of Radiology, Shanghai East Hospital, Tongji University School of Medicine, Shanghai 200120, PR China; 6 Department of Geriatrics, The Sixth People's Hospital of Shanghai, Shanghai Jiao Tong University, Shanghai 200233, PR China

**Keywords:** amnestic mild cognitive impairment, pulsed arterial spin labeling, magnetic resonance imaging, cerebral blood flow, brain compensatory mechanism

## Abstract

We used pulsed arterial spin labeling (PASL) to investigate differences in cerebral blood flow (CBF) between 26 patients with amnestic mild cognitive impairment (aMCI) and 27 controls with normal cognition (NC). Hypoperfusion was observed in the right temporal pole of the middle temporal gyrus and the right inferior temporal gyrus in the aMCI compared with NC group. Interestingly, hyperperfusion was observed in the left temporal pole of the middle temporal gyrus, left superior temporal gyrus, bilateral precuneus, postcentral gyrus, right inferior parietal lobule, and right angular gyrus in the aMCI group, which likely resulted from a compensatory mechanism to maintain advanced neural activities. We found that mean CBF in the right inferior temporal gyrus, precuneus, and postcentral gyrus was positively correlated with cognitive ability in the aMCI but not NC group. Collectively, our data indicate that PASL is a useful noninvasive technique for monitoring changes in CBF and predicting cognitive decline in aMCI.

## INTRODUCTION

Mild cognitive impairment (MCI) is an intermediate stage between normal aging and dementia. Amnestic MCI (aMCI) is characterized by memory impairment and is considered to be a prodromal stage of Alzheimer’s disease (AD) [1]. Approximately 16.5–23% of MCI patients deteriorate to AD annually [2, 3]. There are few sensitive and effective methods for the early diagnosis of AD [4]. Accurate diagnosis and timely intervention in patients with aMCI could reduce the rate of conversion to AD [5].

Arterial spin labeling (ASL) perfusion magnetic resonance imaging (MRI) is widely used to study neurodegenerative diseases [6]. ASL utilizes water in arteries as an endogenous contrast media to enable visualization of tissue perfusion, and can be used to quantify cerebral blood flow (CBF) without the need for radiation or intravenous injection [7]. Reduced CBF has been observed in AD patients, indicating that vascular factors play a critical role in the pathogenesis of the disease [8–13]. Several studies have also reported regional decreases in CBF in MCI patients prior to AD onset [12–15]. However, other studies have observed increases in CBF in the left hippocampus, right amygdala, rostral head of the right caudate nucleus, ventral putamen, and globus pallidus in MCI patients [16]. Thus, there is no consensus regarding abnormal CBF in the MCI stage. Differences in sample size, participants, methodology, and etiology may account for the variable study results [17, 18].

Here, we investigated whether there were differences in CBF between subjects with aMCI and those with normal cognition (NC) using pulsed ASL (PASL). Additionally, we evaluated the relationship between altered CBF and global cognition. Our data indicate PASL can detect early stage cognitive impairment and may be useful for monitoring changes in CBF and predicting cognitive decline in aMCI.

## RESULTS

### Patient characteristics

Patient demographic and neuropsychological data are shown in Table 1. No significant differences in gender, age, education, hypertension, diabetes, stroke, heart disease, or *APOE-ε4* genotype status were observed between the aMCI and NC groups (all P > 0.05). However, a significant difference in the Mini-Mental State Examination (MMSE) score was observed between the two groups (P = 0.016).

**Table 1:** The demographic and clinical characteristics of the patients.

	**MCI (n = 26)**	**NC (n = 27)**	**P value**
Age, mean±SD	73.85±7.40	74.26±6.40	0.829
Gender(M), n(%)	18(69.23)	19(70.37)	0.928
Education, mean±SD	12.00±3.63	12.67±3.56	0.503
Hypertension, n(%)	13(50.00)	11(40.74)	0.498
Diabetes, n(%)	2(7.69)	4(14.81)	0.738
Stroke, n(%)	3(11.54)	0(0.00)	0.221
Heart disease, n(%)	1(3.85)	1(3.70)	1.000
APOE-e4+, n(%)	4(15.38)	5(18.52)	1.000
MMSE, mean±SD	27.35±1.55	28.33±1.33	0.016*

SD: Standard deviation *. Correlation is significant at the 0.05 level (two-tailed).

### Differences in CBF between the aMCI and NC groups

**Figure 1:**
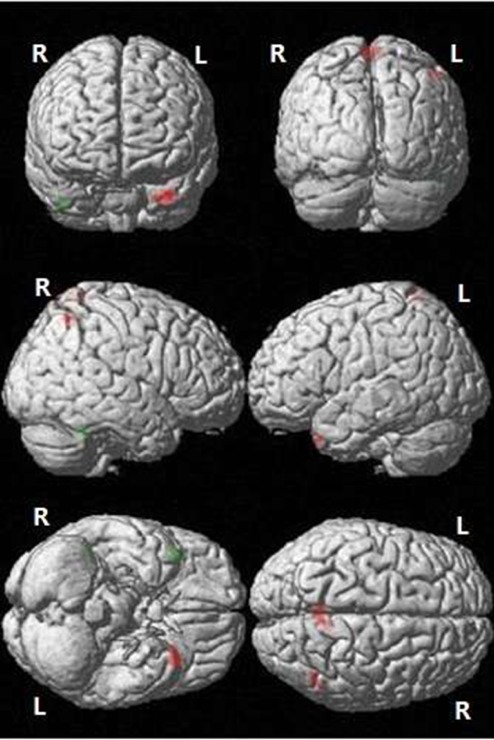
**Differences in CBF between the aMCI and NC groups.** Green: Decreased CBF in the aMCI compared to NC group; Red: Increased CBF in the aMCI compared to NC group (P < 0.05, AlphaSim corrected, cluster size ≥ 76 voxels). R: right; L: left.

Two clusters of hypoperfusion and three clusters of hyperperfusion were observed in the aMCI compared to control group (P < 0.05, AlphaSim corrected, cluster size ≥ 76). Hypoperfusion was observed in the right temporal pole of the middle temporal gyrus and the right inferior temporal gyrus in the aMCI group. Additionally, hyperperfusion in the bilateral precuneus (extending to the postcentral gyrus), left temporal pole of the middle temporal gyrus (extending to the left superior temporal gyrus), and right inferior parietal lobule (extending to the right angular gyrus) was observed in the aMCI group (Figure 1 and Table 2).

**Table 2:** Areas of hypoperfusion and hyperperfusion in the aMCI compared to NC group.

**Cluster**	**Brain area**	**Peak MNI coordinate**			**voxels**	**T value**
		**X**	**Y**	**Z**		
Hypoperfusion						
Cluster1	Right temporal pole of middle temporal gyrus	44	21	-44	112	-4.35
Cluster2	Right inferior temporal gyrus	48	-45	-27	92	-3.27
Hyperperfusion						
Cluster3	Left precuneus (extend to right precuneus, postcentral gyrus)	-5	-54	72	346	4.17
Cluster4	Left temporal pole of middle temporal gyrus (extend to left superior temporal gyrus)	-33	21	-36	312	3.81
Cluster5	Right inferior parietal (extend to right angular gyrus)	47	-60	56	121	3.36

P < 0.05, AlphaSim corrected, cluster size ≥ 76.

### Correlation between abnormal CBF and global cognition

**Figure 2:**
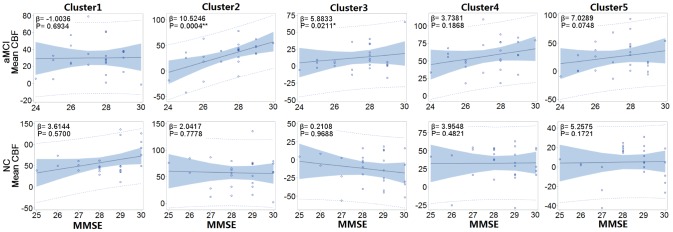
**Correlation between the MMSE and mean CBF of each cluster in each group.** The mean CBFs in right inferior temporal gyrus (Cluster 2), precuneus, and postcentral gyrus (Cluster 3) were positively correlated with MMSE in the aMCI but not NC group. β: Regression parameter; Solid line: line of best fit; Blue shadow: 95% confidence interval; Dashed line: 95% prediction interval. *. Correlation is significant at the 0.05 level (two-tailed). **. Correlation is significant at the 0.01 level (two-tailed).

We next analyzed the relationship between mean CBF and the MMSE score as a measure of global cognition. We adjusted for confounders including gender, age, education, history of hypertension, diabetes, stroke, heart disease, and *APOE-e4* genotype status. A positive correlation between mean CBF in the right inferior temporal gyrus, precuneus, and postcentral gyrus (Clusters 2 and 3) and the MMSE score was observed in the aMCI but not NC group (P < 0.01). No correlation between mean CBF and the MMSE score was observed in the other three clusters (Figure 2).

## DISCUSSION

Perfusion is an important indicator of microvascular distribution, tissue metabolism, and organ function [19]. Detecting changes in CBF is critical for the diagnosis and treatment of neurological disorders. ASL is currently the only non-invasive imaging technique that enables quantitative analysis of CBF [20, 21]. Although positron emission tomography (PET) is considered the gold standard for evaluating CBF, several studies have demonstrated that ASL achieves comparable results [22–25]. ASL is advantageous because it is more economical, faster, safer, and more reproducible than PET. Therefore, it may have strong clinical value.

Recent studies have shown that altered CBF contributes to the pathogenesis of AD [26–31]. For example, de Eulate et al. demonstrated reduced CBF and velocity in patients with cognitive dysfunction, which was correlated with cognitive impairment [32]. Additionally, Riederer et al. observed reduced CBF in global gray matter in AD patients compared to healthy controls, which was consistent with the results of ^18^F-FDG PET [33]. Finally, Shirzadi et al. found that the temporal lobe ASL-spatial coefficient of variation (sCoV) was higher in AD and MCI patients compared to cognitively unimpaired controls [34].

Most studies have demonstrated extensive hypoperfusion in the parietal [10, 12–14, 16, 35], temporal [10, 12, 16, 35], frontal [12, 14, 16], occipital [10, 12], posterior cingulate cortex [9–11, 13–16], precuneus [10, 11, 13, 16, 35], hippocampus and parahippocampal gyrus [9], and left limbic lobe [12] in MCI and AD patients. However, several studies have demonstrated hyperperfusion in the left hippocampus, right amygdala, rostral head of the right caudate nucleus, ventral putamen, and globus pallidus in MCI patients [16], and in the anterior cingulate cortex [16], hippocampus, and other temporal gyrus [35] in AD patients. No significant differences in CBF were observed between aMCI/MCI and cognitively unimpaired individuals in other studies [33, 36, 37]. Discrepancies in regional CBF changes reported by these previous studies may be due to differences in sample size, diagnostic criteria, disease stage, participant heterogeneity, etiology, imaging systems and labeling sequences, study methodology, and therapeutic interventions [17, 18].

Vascular risk factors can increase the risk of MCI and AD [30]. Wierenga et al. demonstrated an interactive effect between cognitive status and APOE genotype on CBF [38]. van der Thiel et al. [39] and De Vis et al. [40] demonstrated that local and global CBF were predictive of cognition among elderly patients during follow-up. Finally, Cecchetti et al. found that brain hemodynamic activity was an intermediate phenotype linking vitamin B12 to cognitive function among elderly patients and predictive of visual search and attention abilities [41]. Thus, monitoring perfusion and timely intervention during the aMCI stage could reduce the risk of progression to AD.

We observed hypoperfusion in the right temporal pole of the middle temporal gyrus and right inferior temporal gyrus, and hyperperfusion in the left temporal pole of the middle temporal gyrus, left superior temporal gyrus, bilateral precunei, postcentral gyri, right inferior parietal lobule, and right angular gyrus in the aMCI group. Mean CBF in the right inferior temporal gyrus, precuneus, and postcentral gyrus was positively correlated with cognitive ability in the aMCI but not NC group. Extensive lesions in the temporal lobe can lead to neurological symptoms such as personality changes, emotional abnormalities, memory impairment, mental retardation, and apathy. Lesions in the parietal lobe can lead to sensory disorders, apraxia, alexia, planotopokinesia, and physical atrophy.

Hypoperfusion in the right temporal pole of the middle temporal gyrus and the right inferior temporal gyrus was observed in aMCI patients in our study. The temporal pole is important for associative memory and comparisons, while the inferior temporal gyrus is important for recognition of three-dimensional objects and differential recognition of two-dimensional graphics. It also plays an important role in color matching and delayed memory. We observed a reduction in CBF in the right temporal pole of the middle temporal gyrus and the right inferior temporal gyrus in the aMCI group. This was indicative of impaired blood oxygen and energy metabolism, which can cause local nerve degeneration. Consistent with these results, Ding et al. demonstrated hypoperfusion in the right middle temporal gyrus and the right inferior temporal gyrus in MCI patients compared to normal controls [12]. We also found that mean CBF in the right inferior temporal gyrus was positively correlated with cognitive level in the aMCI but not control group. These data indicate that the reduction in memory and learning among aMCI patients could result from hypoperfusion in the right inferior temporal gyrus.

Interestingly, we observed hyperperfusion in the temporal lobes of the contralateral dominant hemispheres of the aMCI patients, including the left temporal pole of the middle temporal gyrus and the left superior temporal gyrus. This could be explained by impairment of areas in the right temporal lobe responsible for advanced neural activities in early stage dementia, and could lead to hypoperfusion and a decline in memory and learning ability. The left temporal lobe could promote hyperperfusion as a compensatory mechanism to maintain advanced neural activities by increasing blood oxygen and energy metabolism [42].

We demonstrated that there are areas of hyperperfusion in the parietal lobe including the precuneus, postcentral gyrus, right inferior parietal lobule, and right angular gyrus in aMCI patients. The precuneus has been associated with many high-level cognitive functions such as episodic memory, self-related processing, and consciousness based on functional imaging studies. The postcentral gyrus is a somatosensory area that integrates various stimuli important for the sense of touch. The inferior parietal lobule is associated with the sense of space, mathematical operations, and logic. The angular gyrus is a visual language center that is critical for connecting words and visual and/or auditory images. Impairment of the angular gyrus can result in aphasia, dyslexia, and agraphia.

Previous studies have demonstrated hypoperfusion in the precuneus or lateral parietal cortex in AD patients [8, 10]. Hyperperfusion in the parietal lobe can occur in the aMCI phase as a result of mechanisms in the brain that compensate for altered metabolism. Hypoperfusion occurs when these compensatory mechanisms fail at later stages of the disease process. We found that mean CBF in the precuneus and postcentral gyrus was positively correlated with cognitive level in the aMCI but not control group, which provides further evidence for the existence of a compensatory mechanism during the aMCI phase.

Neuropsychological tests are the main method for the screening and diagnosis of aMCI. However, these tests can be influenced by factors such as the emotional and psychological state of the patients, and educational background. Our results demonstrate that PASL can detect abnormalities in CBF among aMCI patients, suggesting that this technique may provide an objective basis for the diagnosis of aMCI.

Our study had several limitations including the small sample size. However, our study still achieved positive results after AlphaSim correction. Of cause larger sample size may be better in order to obtain more accurate results. Additionally, since we did not include AD patients in the study, our results may only be applicable to aMCI. Finally, we did not perform a longitudinal study and therefore could not evaluate patient prognosis.

Collectively, our findings indicate that monitoring CBF using PASL could facilitate earlier diagnosis and intervention in aMCI. Additionally, mean CBF may be useful for predicting cognitive decline in aMCI patients.

## MATERIALS AND METHODS

### Patient selection

This study was approved by the Medical Ethics Committees of Tongji Hospital, Tongji University and Huashan Hospital, Fudan University, Shanghai, China. All subjects provided written informed consent. Twenty-six individuals with single or multiple domain aMCI, and 27 with NC were recruited from the Jingansi community in Shanghai, China. The inclusion criteria were the following: 1) 60–85 years of age; 2) intact visual, auditory, and speaking abilities, and 3) willingness to undergo neuropsychological tests and MRI. The exclusion criteria were: 1) history (past year) of the following diseases: local brain injury, traumatic brain injury with loss of consciousness, or immediate confusion caused by traumatic brain injury; 2) anxiety, depression, schizophrenia, or mental retardation; 3) alcohol or drug abuse (past year); 4) severely impaired heart, liver, kidney, or lung function; blood disorders; endocrine diseases; neurosyphilis; 5) history of cancer; 6) ≥ 1 lacunar infarction and patchy or diffuse leukoaraiosis as determined by MRI.

### Clinical data collection

The demographic characteristics of the patients including gender, age, and educational background were collected through an interviewer-administered questionnaire. Past medical history including diagnosis with hypertension, diabetes, stroke, or coronary artery disease was obtained from medical records. The following neuropsychological tests were performed: 1) MMSE, 2) Conflicting Instructions Task (Go/No Go Task), 3) Stick Test, 4) Modified Common Objects Sorting Test, 5) Auditory Verbal Learning Test, 6) Modified Fuld Object Memory Evaluation, 7) Trail-making test A&B, and 8) Chinese yuan (official currency of China) Test, which was translated from the EURO Test. Patients with ≥ 6 years of education received tests 1–5 and 7, while subjects with < 6 years of education received tests 1–4, 6, and 8. Normative data and test details are reported elsewhere [43].

All patients received complete medical and neurological exams by professional neurologists. The Center for Epidemiologic Studies Depression and Scale (CESD) guidelines were used to determine whether subjects met the criteria of having a major depressive episode (CESD ≥ 16) within the past week. The Clinical Dementia Rating (CDR) and the Lawton and Brody Activity of Daily Living (ADL) scales were used to assess cognitive complaints, physical self-maintenance ability, and activities of daily living [43].

The consensus diagnosis of MCI or NC was made by an expert panel consisting of two neurologists, one neuropsychologist, and one neuro-epidemiologist based on medical, neuropsychological, and neurological data, and MRI findings. AMCI was defined according to the following criteria [44]: 1) cognitive concern or complaint by the subject, informant, nurse, or physician with a Clinical Dementia Rating (CDR) = 0.5; 2) a deficit in at least one memory test or with an additional deficit in another domain; 3) normal functional activities; and 4) absence of dementia according to the Diagnostic and Statistical Manual of Mental Disorders-IV, DSM-IV.

### Genotyping

DNA was extracted from blood or saliva samples. *APOE* genotyping was performed using the TaqMan SNP genotyping assays. *APOE-ε4* positivity was defined as the presence of one or two ε4 alleles [43].

### Image acquisition

All subjects underwent MRI at Tongji Hospital using a 3.0-T Siemens Verio MR scanner equipped with a 32-channel head coil. Subjects were instructed to stay relaxed, close their eyes, and keep their head still during the scan. Rubber earplugs were used to reduce noise to a minimum and foam pads were placed around the heads to reduce movement.

PASL images were collected using an echo-planar imaging (EPI) sequence with the following parameters: repetition time (TR) = 2,564 ms, echo time (TE) = 11 ms, inversion time (TI) = 700 ms, delay time = 1,800 ms, labeling time = 700 ms, band width = 2,232 Hz / pixel, flip angle (FA) = 90°, thickness = 1.0 mm, field of view (FOV) = 220 mm × 240 mm, matrix = 256 × 256. The overall scan time was 268 s.

Whole brain high-resolution anatomical images were acquired using a 3D magnetization-prepared rapid gradient echo (MPRAGE) T1-weighted sequence with the following parameters: sagittal orientation, TR = 21 ms, TE = 3.6 ms, band width = 186 Hz / pixel, FA = 18°, slice thickness = 0.5 mm, slice = 160, FOV = 200 mm × 180 mm, matrix = 384 × 364. The overall scan time was 265 s.

### Data analysis

The PASL data was processed using the SPM8 software (http://www.fil.ion.ucl.ac.uk/spm) and ASL toolbox (ASLtbx, http://cfn.upenn.edu/~zewang) [45]. The center of each volume was first reset to the origin and all rotations were set to zero. The first PASL image was set as the reference volume and all other images were then motion-corrected relative to the reference. PASL images were realigned relative to the T1-weighted images for each subject. Smoothing of the realigned and coregistered PASL images was performed by applying an SPM Gaussian smoothing kernel. A mask based on the mean of the smoothed PASL images was employed to exclude out-of-brain voxels. Finally, CBF was calculated by subtraction based on the M0csf.

Whole brain CBF in the aMCI and NC groups was compared using two-sample t-tests. P < 0.05 (AlphaSim corrected, using the RESTplus software; http://restfmri.net/forum/RESTplusV1.2) was considered significant. Age and gender were included as covariates in the regression. CBF clusters were visualized using the xjWiew (http://www.alivelearn.net/xjview) toolbox.

Demographic and clinical characteristics were analyzed as follows: continuous variables were expressed as the mean and standard deviation (SD), and were compared using two-sample two-tailed t-tests, while categorical variables were expressed as frequencies (%) and compared between groups using chi-square tests. A generalized linear model (GLM) was used to evaluate the association between mean CBF and MMSE score for each cluster in the aMCI group and NC groups after adjusting for gender, age, education, hypertension, diabetes, stroke, heart disease, and *APOE-ε4* genotype status. P values were estimated using two-tailed tests. A P < 0.05 was considered statistically significant. All data analysis was performed using SAS 9.4 (SAS Institute Inc., Cary, NC, USA).
